# Slovenian validation of the Capacity to Love Inventory: associations with clinical measures and mindfulness

**DOI:** 10.3389/fpsyg.2024.1440013

**Published:** 2024-09-09

**Authors:** Timotej Glavač, Vita Poštuvan, Jim Schmeckenbecher, Nestor D. Kapusta

**Affiliations:** ^1^Department of Psychology, University of Ljubljana, Ljubljana, Slovenia; ^2^Andrej Marušič Institute, Slovene Centre for Suicide Research, University of Primorska, Koper, Slovenia; ^3^Faculty of Mathematics, Natural Sciences and Information Technologies, University of Primorska, Koper, Slovenia; ^4^Department for Psychoanalysis and Psychotherapy, Medical University of Vienna, Vienna, Austria

**Keywords:** capacity to love inventory (CTL-I), personality, mindfulness, psychodynamic perspective, psychometrics properties

## Abstract

**Aim:**

The main purpose of the present study was to validate the Slovenian version of the 41- item Capacity to Love Inventory (CTL-I). Based on psychoanalytic theory, limitations to capacity to love are expected to be associated with personality dysfunction and disintegration as well as fundamental mental capacities such as self-reflection and self-awareness.

**Method:**

To examine these assumptions, a sample of 552 Slovenian non-clinical individuals were recruited through academic networks. The construct validity of the CTL-I was assessed using a confirmatory factor analysis and convergent validity of the CTL-I and its subscales was established against IPO-16, PID-5 BF, MAAS.

**Results:**

Our findings show that the Slovenian version of the CTL-I replicated the six-factor structure, exhibiting good model fit as well as satisfactory internal consistency of all subscales. In line with expectations, capacity to love was found to be inversely associated with dysfunctional personality traits and structural personality disturbances. Accordingly, higher dispositional mindfulness was coherently associated with all domains of CTL-I.

**Conclusion:**

The results add to the growing evidence for the cross-cultural validity and sound psychometric properties of CTL-I, presented here in the Slovenian version. Our findings also point to the significance of dispositional mindfulness both in relation to capacity to love as well as mental health.

## Introduction

Love stands as one of the most gratifying and fundamental of human experiences (Fletcher et al., 2015; Jankowiak and Fischer, 1992; Reis and Downey, 1999). Accordingly, research has consistently demonstrated strong associations between social relationships, particularly romantic ones, and various dimensions of well-being (Diener and Seligman, 2002; Frisch, 2005; Kansky, 2018; Mehl et al., 2010). For example, intimate relationships are linked to heightened subjective well-being (Campbell et al., 2005; Dush and Amato, 2005), while healthy romantic partnerships appear to foster improved physical health and reduced stress reactivity (Coan et al., 2013; Kiecolt-Glaser and Newton, 2001). Moreover, marriage has been shown to promote healthy behaviors among spouses over time (Homish and Leonard, 2008; Meyler et al., 2007), with research suggesting that marital satisfaction holds a stronger association with life satisfaction than domains such as job and health satisfaction (Dobrowolska et al., 2020; Heller et al., 2004). The relationship between life satisfaction and relationship satisfaction seems to be bidirectional in nature (Be et al., 2013).

The ability to form long-lasting committed intimate relationships with another person has been reflected by the concept of capacity to love (CTL) (Kernberg, 1974, 1977, 2011). Capacity to love consists of various elements, it involves the capacity to participate in, invest in, and maintain a dedicated romantic love relationship (Kernberg, 2011). More specifically, these features have been outlined based on clinical observations, and the concept is firmly rooted in psychoanalytic theory, particularly object relations theory (Kernberg, 1974, 1977, 2011; Garza-Guerrero, 2000). In order to be able to assess these constructs quantitatively, Kapusta et al. (2018) designed a 41-item questionnaire, measuring the proposed features of capacity to love. These include, Interest in the other, Basic trust Gratitude, Common ego ideal, Permanence of sexual passion, and Loss and mourning (Kapusta et al., 2018; Kernberg, 2011). Additionally, Falling in love, Forgiveness and Mature dependency, are dimensions which were also proposed in the original theoretical conceptualizations (Kernberg, 2011) but were excluded due to the unsatisfactory psychometric properties of the scales.

**Interest in the other** involves a deep, ongoing curiosity and interest in the emotional experience, personal history, and aspirations of the partner. This is thought to enrich one’s own life and fosters mutual exploration and deeper love, contrasting sharply with the indifference often seen in narcissistic individuals. **Basic trust** in a partner’s empathy and goodwill allows for openness about personal vulnerabilities and needs. This trust enables both partners to reveal themselves, fostering mutual growth and deepening their relationship. **Humility and gratitude** in mature love involve acknowledging dependency and embracing future uncertainties, as well as experiencing a genuine gratitude for the love received and responded to. **Common ego ideal** reflects a couple’s commitment to their love as a lifelong project, influencing daily life and nurturing a deep interest in each other’s personalities and experiences. **Permanence of sexual passion** refers to a passion, continuing throughout the relationship without diminishing after the initial phases, and involves, integrating tenderness with eroticism, supporting a deep connection between partners. Lastly, normal mourning (**Loss and mourning**) strengthens the ability to love and should not be dominated by excessive guilt or self-devaluation and can lead to positive growth after the end of a relationship. Previous validation studies have reported good psychometric properties of the proposed CTL-I model, including adequate construct validity, convergent validity and internal consistency (Kapusta et al., 2018; Margherita et al., 2018).

Derived from clinical psychoanalytic observations (Kernberg, 2011), research initially focused on examining challenges in the capacity to love among individuals with developmental deficits and psychopathology. According to the proposed psychoanalytic framework, narcissistic individuals are most often considered being at risk for experiencing deficiencies in the capacity to love. However, according to Kernberg’s theoretical conceptualizations, the capacity to love is associated with various features of a healthy personality structure, and difficulties in the capacity to love may therefore be expected in various personality disorders as well as varying levels of personality organization (Kernberg, 1974). Indeed, empirical evidence consistently demonstrates that relationship distress serves as a reliable indicator of mental health problems (Whisman et al., 2000).

Difficulties in intimate relationships are one of the most common reasons individuals with acute emotional distress seek treatment (Swindle et al., 2000). Problems in intimate relationships are prevalent among individuals seeking mental health services who otherwise do not report relationship distress as their main cause for treatment (Lin et al., 1996), a finding that has been observed cross-culturally (Foran et al., 2013; Miller et al., 2013). A likely reason for this finding is that interpersonal stress has the potential to produce more intense subjective distress than non-interpersonal stress (Frans et al., 2005). Among mental health diagnoses, personality disorders (PD) may have an especially adverse effect on aspects of romantic relationships and intimate relationship satisfaction (Whisman et al., 2007; Whisman and Schonbrun, 2009). For example, in a study examining the impact of all 10 DSM-IV/DSM-5 Section II Personality disorders (American Psychiatric Association, 2013) on relationship satisfaction. South et al. (2008) found that individuals with PDs were more likely to engage in physical violence and verbal aggression as reported by their partners. PDs have also been shown to be related to higher frequencies of self-reported distress and lower relationship quality (Disney et al., 2012). PDs have also been found to decrease the probability of marriage among histrionic, avoidant and dependent individuals and increase the chances of divorce in paranoid, schizoid, antisocial, histrionic, avoidant, dependent and obsessive-compulsive individuals (Whisman et al., 2007). Similarly, Stroud et al. (2010) collected data on DSM-IV symptoms and found that self-reported paranoid, antisocial, borderline, dependent, and obsessive-compulsive symptoms significantly predicted marital dissatisfaction. A more recent study employing the Personality Inventory for DSM-5 (PID-5; Krueger et al., 2012) in a sample of 52 heterosexual couples found that the strongest effects on relationship dissatisfaction were found in the domains of detachment, negative affect, and disinhibition (DeCuyper et al., 2018). Furthermore, a study employing the DSM-5 found that nearly all the included personality constructs were negatively correlated with both participant-reported and spouse-reported marital satisfaction at each time point in the study (Humbad et al., 2010; South et al., 2008; Stroud et al., 2010).

Lastly, mindfulness, often described as the deliberate practice of focusing on the present moment’s experiences—such as physical sensations, perceptions, emotions, thoughts, and imagery—in a nonjudgmental manner thereby fostering a consistent and nonreactive awareness of these experiences (Grossman et al., 2004; Kabat-Zinn, 1994; Kostanski and Hassed, 2008) has been found to be associated with various aspects of romantic relationships, such as lower relationship conflict, higher sexual satisfaction and increased emotional regulation (see Kozlowski, 2013 for a review). In relation to mental health and personality disorders, mindfulness meditation has been found to ameliorate the symptoms of various personality disorders such as antisocial, avoidant, borderline paranoid, and obsessive-compulsive personality disorders (Shorey et al., 2016; Sng and Janca, 2016; Velotti et al., 2019; Wupperman et al., 2009) as well as symptomatology related to some disorders such as psychosis (Chadwick, 2014; Khoury et al., 2013). Indeed, dispositional mindfulness characterized as a disposition towards a higher frequency of mindful states over time (Brown and Ryan, 2003) has been shown to be inversely related to negative mental health outcomes (Tomlinson et al., 2018) and positively related to psychological well-being (Bränström et al., 2011; Hanley et al., 2015; Soysa et al., 2021).

The main purpose of our study was to expand the process of validation of the CTL-I on a Slovenian translation, as well as to further establish its psychometric properties. The concept of capacity to love shares the same theoretical and clinical basis with Kernberg’s theory of personality organization (Kernberg, 1985, 2009), which convincingly explains how narcissistic and borderline individuals suffer from major difficulties in relationship functioning. We therefore employed both the Personality Inventory for DSM-5 (PID-5-BF; Krueger et al., 2012) and the Inventory of Personality Organization (IPO-16, Zimmermann et al., 2013) to measure convergent validity with the CTL-I. In contrast, the Mindful Attention Awareness Scale (MAAS, Brown and Ryan, 2003) was applied to examine the relation of CTL-I with a theoretically different, but potentially related concept of a psychological trait capacity covering one’s tendency to attend to present-moment experiences in everyday activities, allowing for self-regulation of attention and the none-judgmental acceptance of one’s immediate experiences (Bishop et al., 2004; Kabat-Zinn, 1994). We hypothesized that mindfulness might be beneficial to intimate relationships by enhancing one’s capacity to love, given that mounting evidence has shown that mindfulness and relationship satisfaction appear to be strongly related (Kozlowski, 2013; McGill et al., 2016).

## Method

### Participants

Our research sample consisted of 552 Slovenian non-clinical individuals (112 men and 434 women, 6 other). The individuals who identified as “other” were excluded from the gender specific analyses. The mean age of the sample was 26.03 years (SD = 9.0). Participants were required to be at least 18 years old to participate in the study. The study protocol was approved (038–19-75/2020/4/FFUM) by the ethics committee of the Faculty of Arts, University of Maribor on 31.7.2020 and was conducted under the code of the Helsinki declaration and its subsequent amendments. Our sample was recruited through e-mail invitations sent to several faculties of all three major Slovenian public universities as well as through advertisements on social media. All the psychological measures were fitted into an online survey which the participants could access through a provided hyperlink. The first page of the survey provided a description of the study along with a consent form explaining that starting the survey (clicking a button) was equivalent to providing consent. The relationship status of our sample was as follows: 8.3% married, 45.3% in a relationship, 0.2% widowed, 2.2% divorced, 31.7% single and 34.2% living together with their partner (see Table 1).

**Table 1:** Characteristics of the sample.

Variables	Percentages (Mean ± SD)
Sex	
Men	112
Women	434
Other	6
Age	26.03
Relationship status	
Single	31.7%
Married	8.3%
Non-marital partnership	45.3%
Widowed	0.2%
Divorced	2.2%
Cohabitation	34.2%

### Measures

#### Capacity to love inventory (CTL-I)

The CTL-I is a 41-item measure of six dimensions of the capacity to love. Items are rated on a 4-point scale. The measure consists of six subscales: Interest in the life project of the other (INT) (e.g., “*It is important to me to know the life plan of my partner*,”) Basic trust (BRT) (e.g., “*My weaknesses, inner conflicts and problems are open to the other*,”) Gratitude (e.g., “*I feel gratitude for the existence of my partner*”) (GRT), Common ego ideal (e.g., “*We always try to work on our relationship*” (CEI)), Permanence of sexual passion (e.g., “*Sexual boredom arises in long-term relationships*” reversed) (PSP) and Loss and mourning (e.g., “*I am often unwilling to accept the end of my relationships*”) (LOM). In the original version, the internal reliability of the total scale was 0.90, while the reliability scores for the specific subscales were as follows: INT—0.73, BTR—0.86, GRT—0.81, CEI—0.81, PSP—0.83 and LOM—0.75. In the present study internal reliabilities ranged from 0.67 (INT) to 0.85 (GRT). The inter-scale correlations between INT, BTR, GRT and CEI ranged from 0.57 to 0.79, while inter-scale correlations between PSP and LOM and the other scales were lower and ranged from −0.01 (between LOM and INT) and 0.31 (GRT and PSP) (shown in Table 2). A similar pattern has also been reported in previous CTL-I validation studies (Kapusta et al., 2018; Margherita et al., 2018).

**Table 2:** Correlations between CTL-I subscales.

	INT	BTR	GRT	CEI	PSP	LOM
INT	-	0.57**	0.67**	0.61**	0.23**	−0.01
BTR		–	0.74**	0.68**	0.29**	0.20**
GRT			–	0.79**	0.31**	0.05
CEI				–	0.29**	0.04
PSP					–	0.07
LOM						–
CTL-I total	0.70**	0.80**	0.81**	0.77**	0.64**	0.37**
alpha	0.67	0.79	0.85	0.81	0.80	0.77

**p ≤ 0.01, *N* = 552. INT, Interest; BTR, Basic trust; GRT, Gratitude; CEI, Common ego ideal; PSP, Permanence of sexual passion; LOM, Loss and mourning.

#### Personality inventory for DSM-5 brief form

The PID-5 BF (PID-5-BF; Krueger et al., 2012) is a short 25-items version of the Personality Inventory for DSM-5 with originally 220 items rated on a 4-point Likert scale ranging from 0 (very false or often false) to 3 (very true or often true). The employed short version has shown comparable domain scores to the original version (Bach et al., 2016). The PID-5-BF assesses five key traits of dysfunctional personality proposed in Section III of the DSM-5 (American Psychiatric Association, 2013) consisting of Antagonism (“It’s no big deal if I hurt other peoples’ feelings”), Disinhibition (“People would describe me as reckless.”), Negative affect (“I worry about almost everything.”), Detachment (“I often feel like nothing I do really matters.”) and Psychoticism (“My thoughts often do not make sense to others”). The psychometric properties of the PID-5 BF have been well established in several recent studies (Anderson et al., 2016; Debast et al., 2017; Hopwood et al., 2013). Internal consistencies on the present sample ranged from 0.61 (Disinhibition) to 0.79 (Psychoticism), while the internal consistency of the total scale was 0.86.

#### Inventory of personality organization (IPO-16)

The IPO-16 is a brief version of the IPO (Lenzenweger et al., 2001) measuring the severity of structural impairments in identity (“*I feel that my tastes and opinions are not really my own, but have been borrowed from other people*”), defense (“*It is hard for me to trust people because they so often turn against me or betray me*”), and reality testing (“*I act in ways that appear to others as unpredictable and erratic*”) based on Kernberg’s model of personality (Kernberg and Caligor, 2005). Items are rated on a 5-point Likert scale ranging from 1 = does never apply to 5 = always applies. A previous study of the German version conducted on clinical samples (*N* = 1,300) for the total scale showed good internal consistency at 0.85 (Zimmermann et al., 2013). The internal consistency of the total scale in our sample was 0.87.

#### The mindful attention awareness scale (MAAS)

The MAAS is a 15-item trait measure of one’s tendency to attend to present-moment experiences in everyday activities. The items are self-rated on a scale from one (“almost always”) to six (“almost never”) and assess aspects of mindful experiences such as” *I could be experiencing some emotion and not be conscious of it until sometime later*.” and “*I do jobs or tasks automatically, without being aware of what I’m doing*.” The answers are scored by calculating mean performance across the 15 items. Higher scores reflect greater levels of dispositional mindfulness while lower levels reflect lower levels. The original MAAS validation study showed good internal reliability with an alpha of 0.82 (Brown and Ryan, 2003). The internal reliability of the MAAS on our sample was 0.86.

### Data analysis procedure

The confirmatory factor analysis (CFA) on the Slovenian version of CTL-I was performed using the Maximum Likelihood (ML) as appropriate estimator. Descriptive statistical analyses were performed with the Statistical Package for the Social Sciences (SPSS) 27.0 (IBM Corp, 2020). Confirmatory factor analysis of the CTL-I was carried out in R 4.2.2 (R Core Team, 2022), using lavaan package (Rosseel, 2012). We tested the theory driven model developed by Kapusta et al. (2018) by means of a CFA: a six-factor model with 41 items and scales being allowed to correlate with each other, according to previous examinations (Kapusta et al., 2018; Margherita et al., 2018). Model fit was assessed by means of the fit indexes: (1) the chi-squared (χ2) statistic and its degrees of freedom; (2) the Standardized Root Mean Square Residuals (SRMR); (3) the Root Mean Square Error of Approximation (RMSEA) and its 90% confidence interval (90% CI). In line with Schermelleh-Engel et al.’s (2003) proposed criteria, the model fits the data when χ2/df equal or < 2, RMSEA equal or < 0.05 (90% CI: the lower boundary of the CI should contain zero for exact and be <0.05 for close fit) while Browne and Cudeck (1993) argued that values ranging from 0.05 to 0.08 are indicative of a good adequacy of the model. Additionally, we ran Monte Carlo simulations involving 50 simulated replications to determine if the model had adequate power levels. The model demonstrated adequate fit (χ^2^ = 2544.183, RMSEA = 0.065) and high statistical power for all parameter estimates. Parameter estimates were stable and reliable, with minimal bias and consistent coverage of true values. The simulations showed that power levels of the model were adequate based on the conventional cut-off criteria (Lakens, 2022).

## Results

We tested the theory driven model proposed by Kapusta et al. (2018) by means of a CFA: a six-factor model with 41 items and scales being allowed to correlate with each other (Tables 2, 3). Due to our data following a non-normal distribution we used a robust standard errors optimization with a Satorra-Bentler scaled test statistic. Fit indices were: chi2/degrees of freedom = 2.71 (2070.038/763) SRMR = 0.060, RMSEA = 0.062 (90% CI 0.059–0.065). Based on the results of the χ2 statistic, a lack of overall fit was shown for the model tested (*p* < 0.001), which is likely due to the sensitivity of this statistic to large sample sizes (Hu and Bentler, 1998; Kahn, 2006). In fact, chi-square is highly sensitive to sample size: as the size of the sample increases, absolute differences become a smaller and smaller proportion of the expected value. The larger the sample, the larger and more significant will be the chi squares, even with very small discrepancies among implied and obtained covariance matrices. On the other hand, samples of reduced size may be too prone to accept poor models (Type II error).

**Table 3:** Pearson correlations among study variables.

		Interest	Basic trust	Gratitude	Common ego ideal	Permanence of sexual passion	Loss and mourning	Total scale
MAAS		0.22**	0.33**	0.27**	0.24**	0.22**	0.30**	0.39**
PID-5	Negative affect	−0.02	−0.19**	−0.08	−0.12**	−0.08	−0.45**	−0.25**
	Detachment	−0.25**	−0.39**	−0.36**	−0.29**	−0.20**	−0.29**	−0.43**
	Antagonism	−0.21**	−0.23**	−0.22**	−0.22**	−0.12**	−0.15**	−0.27**
	Disinhibition	−0.18**	−0.25**	−0.25**	−0.22**	0.01	−0.24**	−0.25**
	Psychoticism	−0.20**	−0.29**	−0.22**	−0.16**	−0.11**	−0.32**	−0.32**
IPO-16	Identity	−0.09*	−0.23**	−0.10**	−0.11**	−0.13**	−0.46**	−0.29**
	Reality testing	−0.17**	−0.22**	−0.15**	−0.12**	−0.09*	0.25**	−0.24**
	Primitive defense	−0.17**	−0.30**	−0.24**	−0.21**	−0.11*	−0.31**	−0.32**

** indicates *p* ≤ 0.01, MAAS, Mindful Attention Awareness Scale; PID 5, Personality Inventory for DSM-5; IPO-16, Inventory of Personality Organization 16.

According to other goodness-of fit indices, chi2/degrees of freedom = 3.13 (2598.870/764), SRMR = 0.060, RMSEA = 0.062 (90% CI 0.060–0.065), a good adequacy of the model was shown (Browne and Cudek, 1993; Hu and Bentler, 1998) and was comparable to previous results (Kapusta et al., 2018; Margherita et al., 2018). In order to evaluate the suitability of the proposed six-factor model compared to alternative models, we examined a five-factor model (where we excluded the Permanence of Sexual Passion dimension), as well as a four-factor model (where we also excluded the Loss and Mourning dimension). These two factors were chosen due to their lower convergent validity scores as can be seen in Table 2. The five-factor model chi2/degrees of freedom = 3.47 (2406.250/692), SRMR = 0.061, RMSEA = 0.061 as well as the four-factor model chi2/degrees of freedom = 4.25 (1818.710/428), SRMR = 0.060, RMSEA = 0.077 showed comparable fit indexes to the original model.

**Figure 1:**
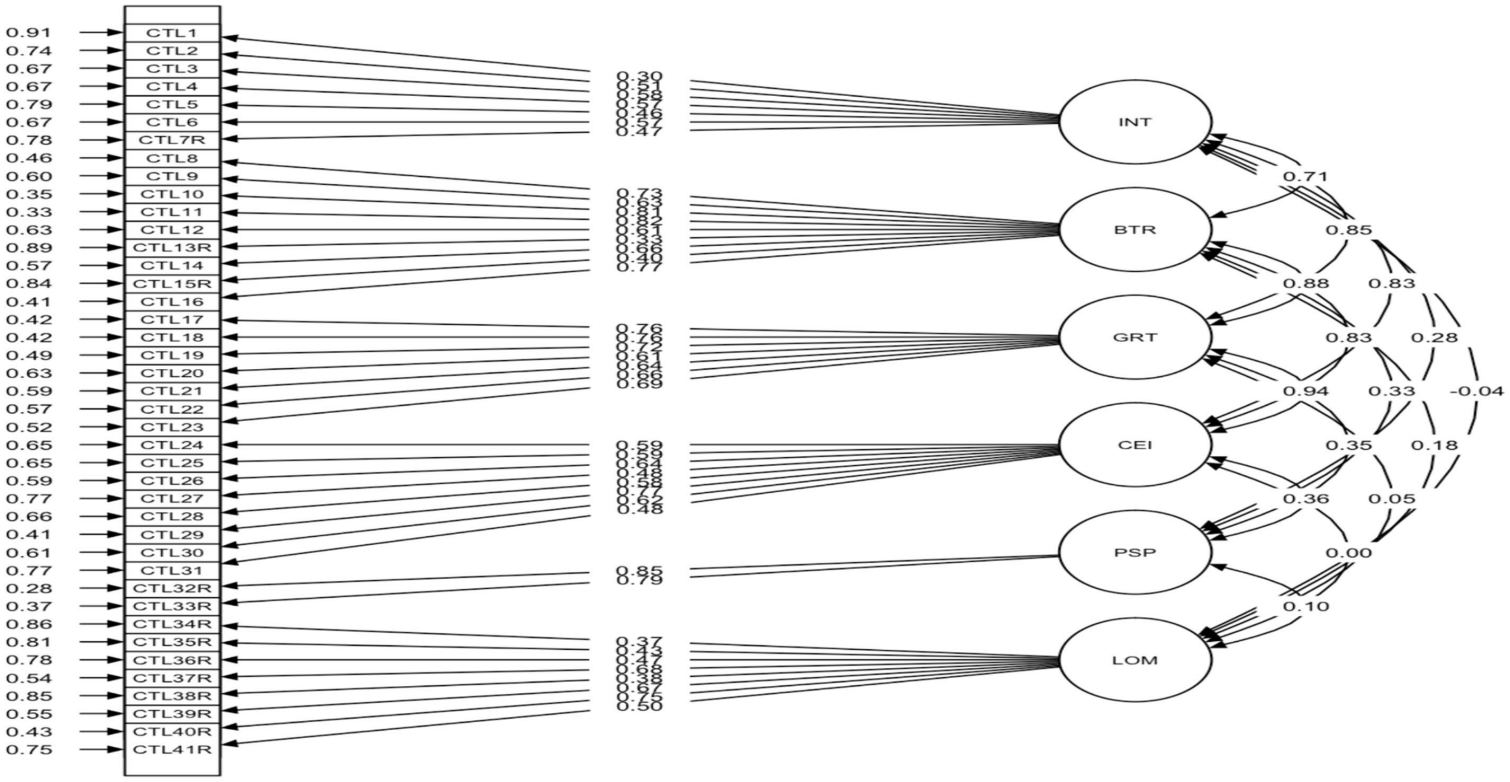
Standardized solution to the CTL model.

To investigate the potential associations among the six CTL-I subscales and other related measures, Pearson correlational coefficients were calculated. The standardized model solution is shown in Figure 1.

Table 2 shows the results of Pearson’s correlation coefficient among the CTL-I subscales. Significant correlations were found among all subscales except for subscale Loss and mourning. The Loss and mourning subscale was significantly correlated with only the Basic Trust subscale and the CTL-I total.

As can be seen in Table 3, Pearson correlations between the PID-5 and CTL-I subscales showed the following results: Negative affect was significantly inversely associated with Basic trust, Common ego ideal, Loss and mourning as well as the total sum score. Detachment, Psychoticism, as well as Antagonism were significantly inversely associated with all CTL-I subscales. Similarly, Disinhibition was significantly inversely correlated with all subscales apart from the Permanence of sexual passion subscale. In examining the relationship between the three IPO subscales with the CTL-I we found that the Identity subscale was significantly inversely correlated with all the CTL-I subscales wherein the strongest association with IPO was between Identity and Loss and mourning as well as the total CTL-I score. Reality testing was also significantly inversely associated with all CTL-I subscales. Finally, the Defense subscale was similarly significantly inversely associated with all CTL-I subscales. Correlations between the Mindful Attention Awareness Scale and the CTL-I showed a strong positive relationship with all CTL-I subscales. In general, these associations fell within the small to moderate range.

To examine the unique contributions of CTL-I dimensions to the included external measures, we conducted hierarchical regression analyses controlling for gender and age in the first step, and then adding the CTL-I dimensions in the second step. The dependent variables included Identity, Primitive defense, Reality testing, Mindfulness, Antagonism, Detachment, Negative affect, Disinhibition and Psychoticism. Results of the hierarchical regressions are presented in Supplementary Tables S1, S2. Due to the covariance between some of the CTL-I scales, several associations observed in the correlations (Table 2) were not observed in the regressions. Overall, these hierarchical regression analyses confirm the robust associations between Basic trust and Loss and Mourning with various external measures, consistent with the presented correlation findings. Other CTL-I dimensions, while correlated, did not uniquely predict the external measures when controlling for other factors, highlighting the unique contributions of these specific facets. The results underscore the contribution of the unique variance of Basic trust and Loss and Mourning in explaining variance in external measures. Furthermore, the results underscore that the CTL-I dimensions explain a substantial amount of the variance in the included dependent variables above what could be seen at the correlational level alone.

Additionally, to explore the potential moderating effects of gender and relationship status on the relationships between external measures and CTL dimensions, we conducted moderation analyses. Before running the moderation analyses, we also examined if homogeneity of variance assumptions were violated in relation to gender and the CTL-I dimensions. Results showed that homogeneity of variances was violated only in Basic Trust: 𝐹 (1,544) = 4.91, 𝑝 = 0.027. Interaction terms were created between the included external measures and the moderator variables (gender and relationship status). Overall, our findings show that in the majority of cases, gender (0—male, 1—female) was not found to be a significant moderator. Except for several cases in relation to Basic trust and the external variables such as (Primitive defense, *p* = 0.026, negative interaction, R^2^ change = 0.0082), Mindfulness, (*p* = 0.050, R^2^ change = 0.0062), Detachment, (*p* = 0.024, negative interaction, R^2^ change = 0.0079), Disinhibition, (*p* = 0.038, negative interaction, R^2^ change = 0.0074), Psychoticism, (*p* = 0.036, negative interaction, R^2^ change = 0.0074) and two cases in relation to Loss and mourning with Primitive defense (*p* = 0.001, negative interaction, R^2^ change = 0.0168) and Disinhibition (*p* = 0.008, negative interaction, R^2^ change = 0.0119) were statistically significant at the more stringent *p* ≤ 01 level. Almost none of the external variables were moderated by relationship status (coded as 0—single, 1—in a relationship), with the exception of Primitive defense in relation to Basic trust (*p* = 0.050, negative, R^2^ change = 0.0058) and Primitive defense in relation to Interest in the other (*p* = 0.050, negative, R^2^ change = 0.0066) as well as Psychoticism in relation to Interest in the other (*p* = 0.045, negative, R^2^ change = 0.0069).

## Discussion

The primary purpose of the present study was to examine the psychometric properties of the Capacity to Love-Inventory in the newly translated Slovenian version of the instrument as well as to examine its external validity with novel, converging measures. So far, the instrument has been validated in German (Kapusta et al., 2018), Polish (Kapusta et al., 2018), Italian (Margherita et al., 2018), Chinese (Li and Dong, 2021) and Slovenian (Poštuvan et al., 2022). To examine the construct validity of the CTL-I we assessed the proposed model by conducting confirmatory factor analysis. A secondary purpose of our study was to examine the relationship between capacity to love and dispositional mindfulness, which is a construct based on a distinctly non-psychoanalytical theory, thus allowing to establish trans-theoretical convergent validity.

Our results confirmed that the Slovenian version of the CTL-I has a clear factor structure and good internal consistency. The proposed six-factor model of the CTL-I demonstrates good fit across various goodness-of-fit indices and maintains theoretical robustness and was found to exhibit superior fit compared to alternative models with fewer factors. Furthermore, fit indexes of the full six-factor model in prior validation studies were very similar to those observed in the present study (Kapusta et al., 2018; Margherita et al., 2018). These results (Kapusta et al., 2018; Margherita et al., 2018) suggest comparable psychometric properties. To examine the convergent validity, we employed the PID-5-BF (Krueger et al., 2012) and the IPO-16 (Zimmermann et al., 2013), measures for assessing personality disorders and personality dysfunction, respectively. We expected both clinical measures to be negatively associated with one’s capacity to love. Our analysis showed that capacity to love was significantly inversely associated to most characteristics of personality dysfunction and traits of personality disorders. These findings suggest that a limited capacity to love may indeed be associated with various forms of psychopathology. Since the capacity to love has previously been examined primarily in relation to a few clinical measures such as depression and narcissism (Kapusta et al., 2018; Margherita et al., 2018), we did not have specific assumptions about the size of these associations. Regardless the findings were in the expected directions as we expected clinical measures to be moderately negatively associated with the capacity to love as deficits in the capacity to love have been considered indicators of psychopathology (Kernberg, 1974, 1977, 2011). While limitations in the capacity to love as well as the construct’s theoretical underpinnings have primarily been associated with narcissism (Kapusta et al., 2018; Kernberg, 2011), our results suggest that noticeable deficiencies of love capacity might be attributable to a wider array of personality features. These findings are in line with studies which have consistently shown personality disorders to be detrimental to relationship quality as well as relationship satisfaction (DeCuyper et al., 2018; South et al., 2020; Whisman et al., 2007; Whisman and Schonbrun, 2009). Relationship distress has been shown to consequentially and bidirectionally influence mental health and life satisfaction (Be et al., 2013). Furthermore, the experiences with significant others are laid down early in life based on interactions with caregivers and the subsequent relationships with romantic partners are likely to partially reflect the nature of these earlier caregiving relationships which are known to be crucial factors in mental health (Kernberg, 2006; Sroufe, 2000). Indeed, issues in romantic relationships as well as social relationships more broadly are commonly associated with mental health difficulties and lower well-being (Diener and Seligman, 2002; Frisch, 2005; Kouros et al., 2008; Groh et al., 2014; Kansky, 2018; Mehl et al., 2010) and our results suggest that a similar pattern might apply for the capacity to love.

We also expected capacity to love to be positively associated with mindfulness as it is a fairly well researched practice that has shown promising results in this regard (Coronado-Montoya et al., 2016: Galante et al., 2021; Kozlowski, 2013). Accordingly, the results of the present study found dispositional mindfulness to be associated with all the capacity to love subscales. In recent years an increasing number of findings show that mindfulness is beneficial to relationship quality (Kozlowski, 2013; McGill et al., 2016). Because the concept of capacity to love originates in a psychoanalytic framework, it is interesting to see mindfulness being positively related to this concept. Due to the assumption of mindfulness being both a disposition as well as a skill which can be practiced, our findings are promising as they seem to suggest that mindfulness practice may improve capacity to love and consequentially relationships satisfaction through changes on a deeper level of personality. The relationship between mindfulness and capacity to love might be a consequence of several factors, including the cultivation of empathy and empathic responding in couples (Block-Lerner et al., 2007), aiding in emotional skillfulness and the consequent benefits in emotional regulation (Wachs and Cordova, 2007) and stress response mitigation (Barnes et al., 2007). Mindfulness training has been shown to increase sexual desire and sexual arousal allowing partners to experience more sexual satisfaction in the relationship (Brotto et al., 2008). Because the present study is cross-sectional in design, causal inferences are not possible, indeed, interventional studies assessing the possible effects of mindfulness training on specific domains of CTL would be needed to test a causal association between both constructs.

Additionally, we examined the unique contribution of the CTL-I scales to the convergent measures. Results of the hierarchical regressions confirm the significant negative associations between specific CTL-I dimensions and various external measures. The findings suggest that the associations observed are not merely due to shared variance among the CTL-I dimensions but reflect the unique predictive power of the dimensions when controlling for other variables. Therein Basic trust and Loss and Mourning were observed to be especially distinct features of capacity to love. A possible explanation for the unique variance of these two dimensions is their similarity to aspects of the attachment process (Bowlby, 1969, 1980), which is known to be an important variable in relationship quality (Collins et al., 2006) as well as mental health and psychosocial functioning more broadly (Ranson and Urichuk, 2008; Zhang et al., 2022). In many ways Basic trust shares similarities with the characteristics of individuals possessing a secure attachment style, such as the capacity to tolerate one’s uncertainty, that sense of security in relationship with others as well as an internalized security as a consequence of positive significant introjects (Kernberg, 2011). Secure attachment has consistently been found to be a vital aspect of relationship quality (Collins et al., 1990; Gleeson and Fitzgerald, 2014; Simpson and Rholes, 2017). The capacity for healthy mourning is a significant factor related to attachment as securely attached individuals are better able to process loss and to mourn important relationships a capacity that is influenced by earlier relationships with important others (Bowlby, 1969, 1980). It is also worth noting that in the original validation study Basic trust and Loss and Mourning had the highest (negative) correlations with pathological narcissism and narcissistic personality traits (Kapusta et al., 2018).

Apart from Basic trust and Loss and Mourning the regression analyses also showed the unique contribution of several other dimensions. For example, Gratitude was a negative predictor of Detachment and Disinhibition. Gratitude is thought to be congruent with a realistic self-regard and a genuine recognition of one’s fundamental need for others in order to attain fuller enjoyment and a sense of security in life. Thus, individuals high in Detachment may struggle to form and maintain the close, emotionally fulfilling relationships that foster a sense of gratitude and Gratitude requires a certain level of emotional engagement and the ability to feel and express positive emotions towards others. Similarly, because Gratitude requires an emotional maturity and recognition of the importance of the other in one’s life, individuals higher in Disinhibition, which is characterized by impulsivity might have bigger difficulties experiencing such Gratitude. Antagonism was also found to be negatively predicted by Common Ego Ideal, which is likely a consequence of altruism and empathy being key features related to the capacity for experiencing common ego ideal and that these are likely thwarted by the predisposition towards hostility in individuals higher in antagonism. Additionally, Permanence of sexual passion was found to contribute unique variance in relationship to mindfulness, a result that might be explained by the previously mentioned finding on the association between mindfulness and sexual desire (Brotto et al., 2008).

Furthermore, in order to examine the potential effects of gender and relationship status we examined additional moderation analyses across the associations. The findings of the moderations indicate that, in the majority of cases, these demographic factors do not significantly alter the relationships between the variables. However, notable exceptions were observed in specific cases. The minimal significant moderation by relationship status suggests robustness and invariance of the CTL dimensions across different relationship statuses. In relation to gender, a few moderation effects were observed in relation to Basic trust and Loss and mourning. This implies that males and females may interpret or experience these constructs slightly differently, which warrants further investigation and potential adjustments to the measurement tool to ensure fairness and accuracy across genders. While measurement invariance could not be examined on our sample, due to the considerable gender imbalance, future research should further consider these additional psychometric properties to better understand the underlying mechanisms and validate these findings across different populations and contexts.

### Limitations

A limitation of our study was a disproportionate sex ratio of our sample—there was a significantly larger number of women who took part in our study. A possible explanation of this finding is that men may tend to be less interested in romantic relationships than women (Fraley et al., 2011) or are at least less interested in taking part in surveys on the topic of romantic relationships. Interestingly, previous CTL-I studies have shown similar results (Kapusta et al., 2018; Margherita et al., 2018). An additional limitation is that due to the significant gender imbalance we were not able to examine measurement invariance in relation to men and women. Furthermore, because our sample included a normative sample of students, higher ranges of psychopathology are not to be expected. Indeed, as the CTL-I validation studies have also been conducted on student samples, results show that the central tendency of the average scores is high (Kapusta et al., 2018; Margherita et al., 2018). Another limitation is the age of our sample, with most of our participants being university students they might have had less relationship experience than their older counterparts. Regardless of age however, most of our sample has had non-negligible relationship experience. Additionally, the moderation analyses showed that relationship status did not significantly influence the relationship between CTL-I dimensions and external measures. Lastly, because the present study was cross-sectional in design no causal inferences can be made on the directionality of dispositional mindfulness and capacity to love.

## Data Availability

The raw data supporting the conclusions of this article will be made available by the authors, without undue reservation.
