# Supplementary material for: Slovenian validation of the Capacity to Love Inventory: associations with clinical measures and mindfulness
Source: Front Psychol. 2024 Sep 9;15:1440013. doi: 10.3389/fpsyg.2024.1440013 (PMC11417625; doi:10.3389/fpsyg.2024.1440013)
Supplement: Supplementary file 1 [file Data_Sheet_1.docx]

Table S1*.* Hierarchical regression analysis predicting the five dimensions of the personality disorder inventory from demographic variables and facets of the capacity to love

| Predictor | NA | | | DE | | | AN | | | DI | | | PS | | |
| --- | --- | --- | --- | --- | --- | --- | --- | --- | --- | --- | --- | --- | --- | --- | --- |
|  | B | SE | β | B | SE | β | B | SE | β | B | SE | β | B | SE | β |
| Block 1 | R^2^ = . 0.05** | | | R^2^ = .0.01 | | | R^2^ = .021** | | | R^2^ = 0.02** | | | R^2^ = 0.02** | | |
| Gender | -.35 | .07 | -.09** | -.11 | .05 | -.09* | -.10 | .04 | -.09* | -.17 | .05 | -.14** | -.11 | .06 | -.07 |
| Age | -.00 | .00 | -.06* | .00 | .00 | -.00 | -.00 | .00 | -.10* | -.00 | .00 | -.04 | -.00 | .00 | -.10 |
| Block 2 | R^2^ = 0.21** | | | R^2^ = 0.23** | | | R^2^ = .097** | | | R^2^ = 0.14** | | | R^2^ = .17** | | |
| INT | .13 | .10 | -.07* | .05 | .08 | .03 | -.08 | .07 | -.07 | .04 | .08 | .03 | -.13 | .10 | -.07 |
| BRT | -.12 | .09 | -.09 | -.21 | .07 | -.19** | -.09 | .06 | -.10 | -.07 | .06 | -.07 | -.26 | .09 | -.19** |
| GRT | .07 | .10 | .05 | -.23 | .08 | -.21** | .01 | .07 | .01 | -.16 | .08 | -.16* | -.11 | .10 | -.08 |
| CEI | -.19 | .10 | -.12 | -.00 | .08 | -.00 | -.14 | .07 | -.14* | -.12 | .07 | -.11 | .11 | .10 | .08 |
| PSP | -.03 | .03 | -.04 | -.06 | .03 | -.10* | -.03 | .02 | -.06 | .05 | .03 | .09* | -.04 | .03 | -.05 |
| LOM | -.48 | .05 | -.42** | -.20 | .04 | -.23** | -.09 | .03 | -.11* | -.19 | .03 | -.23** | -.30 | .04 | -.27** |
| Adj. total R^2^ | R^2^ = 0.25 | | | R^2^ = .0.23 | | | R^2^ = 0.10 | | | R^2^ = 0.15 | | | R^2^= 0.18 | | |

*Note*. ** *p* < 0.01; **p* < 0.05,  *N = 552. INT – Interest, BTR – Basic trust, GRT – Gratitude, - CEI – Common ego ideal, PSP – Permanence of sexual passion, LOM – Loss and mourning, NA – Negative affect, DE – Detachment, AN – Antagonism, DI – Disinhibition, PS – Psychoticism.*

| Predictor | MAAS | | | ID | | | RT | | | PD | | |
| --- | --- | --- | --- | --- | --- | --- | --- | --- | --- | --- | --- | --- |
|  | B | SE | β | B | SE | β | B | SE | β | B | SE | β |
| Block 1 | R^2^ = .001 | | | R^2^ = .03** | | | R^2^ = .03 | | | R^2^ = .02** | | |
| Gender | .14 | .08 | .08 | .08 | .07 | .05 | -.19 | .07 | -.11** | -.18 | .07 | -.10* |
| Age | .00 | .00 | .02 | -.01 | .00 | -.16** | -.01 | .00 | -.13** | -.00 | .00 | -.09* |
| Block 2 | R^2^ = .21** | | | R^2^ = 24** | | | R^2^ = .11** | | | R^2^ = .17** | | |
| INT | .03 | .12 | .02 | -.01 | .11 | -.00 | -.19 | .11 | -.09 | .06 | .11 | .03 |
| BRT | .28 | .10 | .18** | -.26 | .09 | -.17** | -.26 | .10 | -.18** | -.28 | .09 | -.19** |
| GRT | .10 | .12 | .07 | .14 | .11 | .10 | .06 | .11 | .04 | -.07 | .11 | -.05 |
| CEI | .05 | .12 | .03 | -.09 | .10 | -.06 | .02 | .11 | .01 | -.12 | .11 | -.07 |
| PSP | .14 | .04 | .15** | -.09 | .04 | -.10 | -.05 | .04 | -.06 | -.03 | .04 | -.04 |
| LOM | .33 | .05 | .25** | -.49 | .05 | -.41** | -.26 | .05 | -.21** | -.31 | .05 | -.26** |
| Adj. total R^2^ | R^2^ = .20 | | | R^2^ = .25 | | | R^2^ = .12** | | | R^2^ = .18 | | |

Table S2*.* Hierarchical regression analysis predicting the three dimensions of personality organization from demographic variables and facets of the capacity to love

*Note*. ** *p* < 0.01; **p* < 0.05,  *N = 552. INT – Interest, BTR – Basic trust, GRT – Gratitude, - CEI – Common ego ideal, PSP – Permanence of sexual passion, LOM – Loss and mourning, ID – Identity, RT – Reality testing, PD – Primitive defense.*

S3. Results of the significant moderation effects of gender and relationship status on the relationship between the CTL-I dimensions and the included variables

| **Dependent Variable** | **Independent Variable** | **Moderator** | **Interaction** | β | **SE** | **p-value** | **R² Change** |
| --- | --- | --- | --- | --- | --- | --- | --- |
| Basic Trust | Primitive Defense | Gender | Primitive Defense* Gender | -0.15 | 0.07 | 0.026 | 0.0082 |
| Basic Trust | Mindfulness | Gender | Mindfulness * Gender | 0.12 | 0.06 | 0.05 | 0.0062 |
| Basic Trust | Detachment | Gender | Detachment * Gender | -0.14 | 0.06 | 0.024 | 0.0079 |
| Basic Trust | Disinhibition | Gender | Disinhibition * Gender | -0.13 | 0.06 | 0.038 | 0.0074 |
| Basic Trust | Psychoticism | Gender | Psychoticism * Gender | -0.13 | 0.06 | 0.036 | 0.0074 |
| Loss and Mourning | Primitive Defense | Gender | Primitive Defense * Gender | -0.22 | 0.06 | 0.001 | 0.0168 |
| Loss and Mourning | Disinhibition | Gender | Disinhibition * Gender | -0.17 | 0.06 | 0.008 | 0.0119 |
| Basic Trust | Primitive Defense | Relationship Status | Primitive Defense * RS | -0.12 | 0.06 | 0.05 | 0.0058 |
| Interest in the Other | Primitive Defense | Relationship Status | Primitive Defense * RS | -0.14 | 0.07 | 0.05 | 0.0066 |
| Interest in the Other | Psychoticism | Relationship Status | Psychoticism * RS | -0.15 | 0.07 | 0.045 | 0.0069 |

*Note*. ** *p* < 0.01; **p* < 0.05, RS *–* Relationship status, SE *–* Standard error.
